# Evaluating the comprehensive diagnosis efficiency of lung cancer, including measurement of SHOX2 and RASSF1A gene methylation

**DOI:** 10.1186/s12885-024-12022-1

**Published:** 2024-03-02

**Authors:** Jian Liu, Tingting Bian, Bin She, Lei Liu, Hui Sun, Qing Zhang, Jun Zhu, Jianguo Zhang, Yifei Liu

**Affiliations:** 1 Department of Oncology, Affiliated Hospital of Nantong University, Nantong, 226001 China; 2 Department of Pathology, Affiliated Hospital of Nantong University, No 20, Xisi Road, Nantong, 226001 China; 3 Medical School of Nantong University, Nantong, 226001 China; 4 Academic Development, Shanghai Methyldia Technology Co. Ltd, Tellgen Corporation, No. 412 Huiqing Road, Shanghai, 201203 China; 5 Department of Cardio-Thoracic Surgery, Affiliated Hospital of Nantong University, Nantong, 226001 China

**Keywords:** Lung cancer, DNA methylation, SHOX2, RASSF1A, Diagnosis

## Abstract

Methylation of the promoters of SHOX2 and RASSF1A (LungMe®) exhibits promise as a potential molecular biomarker for diagnosing lung cancer. This study sought to assess the aberrant methylation of SHOX2 and RASSF1A in broncho-exfoliated cells (BEC) and compare it with conventional cytology, histology examination, immunohistochemistry, and serum tumor markers to evaluate the overall diagnostic efficiency for lung cancer. This study recruited 240 patients, including 185 malignant cases and 55 benign cases. In our observation, we noted a slight reduction in the detection sensitivity, however, the ΔCt method exhibited a significant enhancement in specificity when compared to Ct judgment. Consequently, the ΔCt method proves to be a more appropriate approach for interpreting methylation results. The diagnostic sensitivity of cytology and histology was in ranged from 20.0%-35.1% and 42.9%-80%, respectively, while the positive detection rate of LungMe® methylation ranged from 70.0% to 100%. Additionally, our findings indicate a higher prevalence of SHOX2( +) among patients exhibiting medium and high expression of Ki67 (*P* < 0.01), as opposed to those with low expression of Ki67, but RASSF1A methylation did not show this phenomenon (*P* = 0.35). Furthermore, CEA, SCCA, and CYFRA21-1 showed positive detection rates of 48.8%, 26.2%, and 55.8%, respectively. Finally, we present a comprehensive lung cancer diagnostic work-up, including LumgMe® methylation. The combined analysis of SHOX2 and RASSF1A methylation serves as a powerful complement and extension to conventional methods, enhancing the accuracy of a lung cancer diagnosis with satisfactory sensitivity and specificity.

## Introduction

Lung cancer is the leading cause of cancer-related deaths of worldwide, accounting for nearly 30% of all cancer deaths in China [1]. Despite advancements in new therapeutic agents such as molecular targeting drugs and immune checkpoint inhibitors, the 5-year survival rate for patients diagnosed at an advanced stage remains challenging to improve [2]. Early diagnosis plays a crucial role in improving lung cancer survival rates, and the widespread implementation of low-dose chest computed tomography (LDCT) screening is expected to have a significant impact on both the incidence and mortality of lung cancer. Recent meta-analyses, including nine studies evaluating the efficacy of LDCT screening on lung cancer outcomes, showed that LDCT screening significantly reduced lung cancer mortality, although not overall mortality. The evidence strongly suggests that LDCT screening nearly doubles the likelihood of diagnosing stage I lung cancer compared to conventional care (48.5% & 24.3%) [3]. However, 24% of LDCT results are abnormal, with 96.4% of these being false positives and an over-diagnosis rate of approximately 18% [4]. This high false positive rate necessitates additional screening rounds or invasive diagnostic follow-ups to confirm the results. Therefore, while LDCT may be sensitive in screening small pulmonary nodules and identifying high-risk populations for lung cancer, there is a requirement to improve the accuracy of lung cancer diagnosis to reduce morbidity and healthcare costs associated with false positives.

Lung cancer is primarily diagnosed using bronchoscopy and transthoracic aspiration. During bronchoscopy, bronchial or bronchoalveolar lavage fluid (BALF) is obtained through a routine lung irrigation technique. The collection of BALF requires a minimally invasive procedure that can be repeated with minimum risk. Moreover, due to its proximity to neoplastic tissue, BALF could exhibit higher sensitivity for biomarker detection compared to other body fluids, such as plasma or pleural fluid [5]. Conventional BEC cytology alone has limited sensitivity for diagnosing lung cancer ranging from 29 to 69%. Although combined morphological techniques, such as the ThinPrep cytology test (TCT), DNA ploidy analysis, and immunohistochemistry, appear to increase the diagnostic yield, they still have some diagnostic gray areas that cannot be resolved by cytopathologists [6, 7]. With the introduction of novel molecular techniques, a broad range of genomic, epigenomic, and transcriptomic tests with higher sensitivity can be performed by amplification of nucleic acids extracted from cells or cell-free forms in BEC. DNA methylation markers are emerging biomarkers and are frequently employed for early cancer diagnosis or recurrence. The tumor cells genome exhibits widespread or localized hypermethylation, particularly within the promoter CpG islands of tumor suppressor genes. Methylation occurring within these promoter CpG islands has the potential to induce transcriptional silencing of the corresponding genes, a majority of which are tumor suppressor genes (TSGs), thereby playing a role in the development of oncogenesis [8]. The LungMe® assay is a powerful and practical diagnostic technique that detects aberrant DNA methylation of the SHOX2 and RASSF1A gene locus. Ras association domain-containing protein 1A (RASSF1A) is a well-researched tumor suppressor gene (TSG) that plays a significant role in cell proliferation, tumorigenesis, and migration. The deficiency of RASSF1A, which results in the activation of YAP, is a crucial factor in the acquisition of a malignant phenotype, invasive and antiapoptotic properties, and eventual transformation into cancer cells by bronchial epithelial cells [9]. Short stature homeobox 2 (SHOX2), situated on chromosome 3q25.32, belongs to the paired homeobox gene family, and its absence of expression leads to the manifestation of short stature syndrome. SHOX2 serves as a regulator of cellular proliferation and apoptosis, as well as an inducer of epithelial-mesenchymal transition (EMT) [10]. Moreover, SHOX2 exerts inhibitory effects on the Hippo-YAP signaling pathway through the activation of NPHP4, thereby facilitating the proliferation and metastasis of prostate cancer [11]. SHOX2 directly influences the activation of the metastasis-promoting gene WASF3 at the transcriptional level, resulting in a notable augmentation of metastatic capability. In terms of mechanism, SHOX2 triggers the activation of STAT3 and facilitates its recruitment to the WASF3 promoter. In this context, STAT3 collaborates with SHOX2 to form a functional immunocomplex, thereby enhancing the transcriptional activity of WASF3 in breast cancer cells and promoting breast cancer metastasis [12]. It has been reported that combining SHOX2 and RASSF1A methylation in BALF achieved a diagnostic sensitivity of 71.5–81.0% and a specificity of 90–97.4% [13, 14]. In this study, we describe the integration of this new technology into our routine pathological detection process and evaluate the diagnostic efficiency of SHOX2 and RASSF1A methylation in broncho-exfoliated cells, comparing it with conventional cytology TCT examination, histology, immunohistochemistry, and serum tumor markers. Finally, we discuss how to integrate different test results to provide a more comprehensive and conclusive pathological diagnosis report.

## Materials and methods

### Patients and specimens

This study was approved by the ethics committee of the Affiliated Hospital of Nantong University with the registration number 2021-L021. All samples were collected from consenting individuals according to protocols approved by the ethics committee. The patients/participants provided written informed consent to participate in this study.

Between September 2021 and May 2022, patients with lung diseases were selected as study participants from the Affiliated Hospital of Nantong University. A total of 240 exfoliated cell samples were obtained through bronchoscopy, including 185 cases of lung cancer, 104 lung adenocarcinoma (LUAC), 53 lung squamous cell carcinoma (LUSC), 23 small-cell lung carcinomas (SCLC), and 5 other metastatic cancers as well as 55 cases of benign lung diseases (37 inflammatory pseudo-tumors, 6 tuberculosis, and 12 other benign diseases). Among the lung cancer cases, 28 were in stage I, 20 in stage II, 40 in stage III, and 97 in stage IV. The distribution of tumor size (largest diameter of pulmonary nodule) was observed across various ranges. Among the cases analyzed, 10 cases in the range of 0–10 mm, 124 cases in 11–50 mm, 29 cases in 51–100 mm, and 12 cases in 101–300 mm. The smoking rate was 3.3% among women and 41.9% among men. The baseline characteristics of patients are shown in Table 1.

**Table 1:** Baseline characteristics of patients

	Lung cancer		Benign disease	
	(185)		(55)	
	n	%	n	%
Age (years)				
Mean ± SEM	66.8 ± 9.2		65.0 ± 10.8	
Range	32–85		19–85	
Gender				
Female (%)	61	33.00%	17	29.10%
smorking	2	3.30%	0	0%
Male (%)	124	67.00%	38	70.90%
smorking	52	41.90%	19	50%
Histology subtype				
LUAC	104	56.20%	-	
LUSC	53	28.60%	-	
SCLC	23	12.40%	-	
Metastatic Cancer	5	2.70%	-	
Infalmmatory Pseudotumors			37	67.30%
Pulmonary Tuberculosis			6	10.90%
Others benign disease			12	21.80%
Stages of Lung cancer				
I	28	15.10%	-	
II	20	10.80%	-	
III	40	21.60%	-	
IV	97	52.40%	-	
Largest diameter of pulmonary nodule				
0 ~ 10 mm	10	5.40%	-	
11 ~ 50 mm	124	67.00%	-	
51 ~ 100 mm	29	15.70%	-	
101 ~ 300 mm	12	6.50%	-	
Unknown	10	5.40%	-	

*Abbreviation*: *LUAC* Lung adenocarcinoma, *LUSC* Lung squamous carcinoma, *SCLC* Small Cell Lung Carcinoma

### DNA extraction and bisulfite treatment

DNA extraction and bisulfite conversion of the cell pellets were performed using the Methy-All-In-One Kit (Tellgen Co., Shanghai, China), following previously described methods [15].

For DNA extraction, precipitation of 5–10 mL of BEC was carried out by centrifugation at 10,000 rpm for 5 min. The concentration of the extracted DNA was accurately measured using highly sensitive fluorescent dye assays (Fluo-100B, Hangzhou Allsheng Instruments Co., Ltd., China). Two hundred nanograms of DNA per sample were subjected to sodium bisulfite treatment using the Tellgen DNA Purification Kit (PF03X056, Tellgen Co., China). This technique involves treating methylated DNA with bisulfite, which converts unmethylated cytosines into uracil while leaving methylated cytosines unaffected during the treatment.

### SHOX2 and RASSF1A methylation analysis

During the bronchoscopic examination, various cytological specimens can be obtained, including brushing, selective bronchial lavage, curettage, transbronchial needle aspiration, rinse fluids of the forceps, and all aspirated fluids. We referred to all these cell samples as “broncho-exfoliated cells (BEC)”. This study disregards the sampling method and instead concentrates on examining the disparities in methylation, cytology, and histology within a single bronchoscopic sample. The objective is to enhance the precision of pathological reporting by employing a combination of multiple detection techniques.

The levels of SHOX2 and RASSF1A DNA methylation in BEC samples were determined using the LungMe® assay, an in vitro diagnostic (IVD) test marked by the China National Medical Products Administration (NMPA) (20,173,403,354, Tellgen Co., China). After purification, the bisulfite-converted DNA was subjected to methylation-specific real-time PCR (MA-PCR) amplification using the LungMe® real-time PCR Kit, as previously reported [13, 14]. The MS-PCR amplifies methylated SHOX2 (VIC), RASSF1A (FAM), and β-ACTB (CY5) DNA, which served as an internal control for quantifying the total input DNA. Positive quality controls were plasmids containing methylated DNA of SHOX2 and RASSF1A without bioactivity. PCR amplification was performed using a HONGSHI SLAN-48 s Real-Time PCR instrument, and SDS Software (Shanghai Hongsi Medical Technology Co. Ltd) was used for result analysis.

The primer and probe sequences used were as follows: the forward primer of SHOX2: TTGTTTTTGGGTTCGGGTT, the reverse primer of SHOX2: CATAACGTAAACGCCTATACTCG, the probe of SHOX2: VIC-ATCGAACAAACGA AACGAAAATTACC, the forward primer of RASSF1A: CGGGGTTCGTTTTGTGGTTTC, the reverse primer of RASSF1A: CCGATTAAATCCGTACTTCGC, and the probe of RASSF1A: FAM-TCGCGTTTGTTAGCGTTTAAAGT.

For the problem of tumor cell proportion, ΔCt = Ct_gene_ – Ct_β-ACTB_. The Ct_β-ACTB_ encompasses the entirety of cell DNA, while Ct serves as an approximate indicator of tumor cell proportion within the overall cell population. Notably, a higher concentration of tumor cells corresponds to a lower Ct value.

Samples were included in the analysis when 18 ≤ Ct_β-ACTB_ ≤ 30. Methylation levels of the gene of interest were expressed as ΔCt, where ΔCt = Ct (gene of interest)—Ct (internal control).

### Receiver Operating Characteristic (ROC) analysis

The receiver operating characteristic (ROC) curve was constructed using Ct values of SHOX2 and RASSF1A methylation as independent variables, while the pathological diagnosis of benign and malignant bronchoalveolar lavage fluid served as the dependent variable. The Logistic regression curve was employed to forecast the likelihood of SHOX2 and RASSF1A methylation in diagnosing bronchoalveolar lavage fluid. Subsequently, the ROC curve was utilized to compute the Area Under the Curve (AUC) for assessing the impact of SHOX2 and RASSF1A methylation combined in the diagnosis of lung cancer.

### Cytological analysis

The BEC solution (10–20 ml) underwent centrifugation at a speed of 404 × g for 10 min, after which the supernatant was discarded. The resulting precipitate was resuspended in 20–25 mL of distilled water and subjected to a subsequent centrifugation at the same speed and duration, with the supernatant being discarded. The obtained precipitate was used to prepare ethanol-fixed glass slides, which were stained with Papanicolaou for cytological examination. The diagnostic report was issued by the senior pathologist affiliated with the hospital. To preserve the bronchial exfoliated cells, the precipitate was immediately transferred to a preservation solution and thoroughly brushed for preservation. When prepared for further processing, the cells were centrifuged at 1500–2000 r/min for 5–10 min. The supernatant was discarded, and 20–30 mL of washing solution was added. The solution was then shaken (1500–2000r/min) for 10 min, subjected to another round of centrifugation at a speed of 1500–2000r/min for 5–10 min, and the supernatant was discarded. The sediment was then poured into a sample bottle containing Thinprep preservation solution and left for 15 min. Subsequently, the Thinprep 2000 system was used for computer programming to prepare a thin layer of cell smear with a diameter of 20 mm. The smear was fixed with 95% ethanol for 10 min for HE staining or Pasteur cell staining, enabling microscopic examination.

### Histopathology analysis

For the HE staining method, the sections that had been placed in distilled water were immersed in an aqueous solution of hematoxylin and stained for several minutes. Then, acid and ammonia water were used for color separation, each for a few seconds. The sections were rinsed with running water for 1 h and then soaked in distilled water for a short period. Next, sections were dehydrated in 70% and 90% alcohol for 10 min each. The sections were stained for 2–3 min in an alcohol eosin staining solution. After staining, the sections were dehydrated using pure alcohol and made transparent using xylene. The transparent section was dripped with gum and sealed with a cover glass. The stained situation was evaluated independently by three experienced pathologists using a multihead microscope (Precise Instrument, Beijing, China).

### Immunohistochemical (IHC) detection interpretation

The IHC analyses were conducted using specific antibodies. Rabbit anti-human polyclonal antibodies against Ki-67 (Thermo Fisher Scientific, catalog # MA5–14,520, RRID AB_10979488), Napsin A (Thermo Fisher Scientific, catalog # PA5–60,970, RRID AB_2644471), TTF-1 (Thermo Fisher Scientific, catalog#PA5–78,209, RRID AB_2736758), CK5/6, P63, and P40 (Roche, Switzerland) were utilized as primary antibodies. The goat anti-rabbit polyclonal antibody was used as the secondary antibody. IHC was performed on a Ventana BenchMark Ultra system. Positive control sections with known positive staining were included, while sections treated with PBS instead of primary antibody served as negative controls. The IHC results were evaluated based on the staining intensity and percentage of tumor cells showing positive staining. For Napsin A, TTF-1, CK5/6, P63, and P40, the results were interpreted as positive or negative based on the staining intensity. Ki-67 IHC results were based on the percentage of positive cells: a percentage of positive cells ≤ 10% indicated a low Ki-67 expression; a percentage between 10–60% indicated a medium Ki-67 expression, and a percentage of ≥ 60% indicated a high Ki-67 expression [16].

### Serum tumor markers

A total of 3 mL of venous blood was collected to obtain serum through centrifugation at 404 × g for 10 min, with the purpose of quantitatively detecting multiple tumor markers. According to the manufacturer's instructions, the fluorescence signal was captured using a chemiluminescence immunoassay kit (Abbott, ARCHITECT i4000SR System). The critical values for the markers were as follows: CEA > 5.0 ng/mL, SCCA > 1.5 ng/mL, and CYFRA21-1 > 3.3 ng/mL. Serum samples were analyzed immediately.

### Statistical analysis

Statistical analysis was performed using SPSS 26.0 and GraphPad Prism 8.4.0. The final clinical and pathological diagnosis was considered the gold standard. The χ2 test was used to assess differences between groups. The performance of each index and the combination of multiple indexes were evaluated using the receiver operating characteristic curve (ROC) curve, and the area under the curve (AUC) values were calculated. A *p*-value less than 0.05 was considered statistically significant.

## Results

### Overview of the enrolled population

**Fig. 1:**
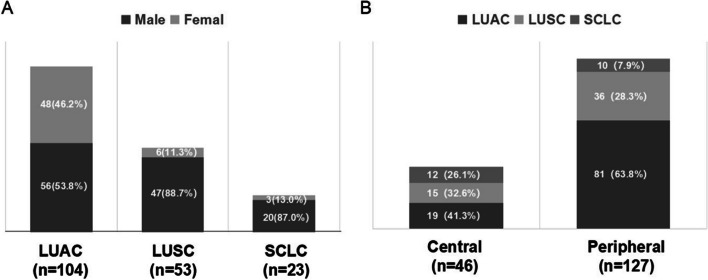
**A** Correlation between sex and pathological classification of lung cancer. **B** Correlation between nodule location and pathological classification of lung cancer

The proportion of men was higher than that of women in both lung cancer and benign lung diseases. The smoking rate among women was 3.3%, while among men it was 41.9% (Table 1). Further analysis revealed that the proportion of men in LUSC (47/53, 88.7%) and SCLC (20/23, 87.0%) was significantly higher than in LUAC (56/104, 53.8%) (Fig. 1A).

Among exfoliated cell samples obtained through bronchoscopy, the most prevalent cancer type was LUAC (56.2%). Furtherly, the lung cancer was categorized into central type (46/173) and peripheral type (127/173) based on the tumor location. The proportion of LUSC and SCLC was higher in central lung cancer compared to peripheral lung cancer (Fig. 1B).

### ΔCt method improves the specificity of methylation detection

In previous studies, the interpretation of qPCR followed the manufacturer’s instructions. A threshold cycle (Ct) value of less than 32 was considered a positive for SHOX2 methylation [SHOX2( +)], while a threshold cycle (Ct) value of less than 35 was considered positive for RASSF1A methylation [RASSF1A ( +)] [13, 14]. In our practical work, we employed the ΔCt method to interpret the relative amount of methylated SHOX2 and RASSF1A, calculated as ΔCt_SHOX2_ = Ct_SHOX2_ − Ct_β-ACTB_ and ΔCt_RASSF1A_ = Ct_RASSF1A_ − Ct_β-ACTB_. We observed that although the ΔCt method slightly reduced sensitivity, it significantly improved the specificity in methylation detection. Furthermore, the ΔCt methylation results remained highly consistent with the clinical diagnosis (Table 2). Based on these findings, we determined that the ΔCt method is a more suitable approach for interpreting methylation results in our subsequent studies.

**Table 2:** Compare the diagnostic sensitivity and specificity of methylation based on Ct or ΔCt judgment

Cutoff value	SHOX2 ( +)		RASSF1A ( +)		LungMe® ( +)	
	Ct < 32	Ct < 32 and ΔCt ≤ 9	Ct < 35	Ct < 35 and ΔCt ≤ 12	Ct_SHOX2_ < 32 or Ct_RASSF1A_ < 35	Ct_SHOX2_ < 32 and ΔCt_SHOX2_ ≤ 9 or Ct_RASSF1A_ < 35 and ΔCt_RASSF1A_ ≤ 12
Sensitivity (*n* = 185)	88.1%	78.4%	48.1%	45.9%	94.1%	87.6%
Specificity (*n* = 55)	83.6%	94.5%	87.3%	96.4%	72.7%	90.9%
consistency (*n* = 240)	87.1%	82.1%	57.1%	57.5%	89.2%	88.3%

### Quantitative analysis of SHOX2 and RASSF1A DNA methylation

**Fig. 2:**
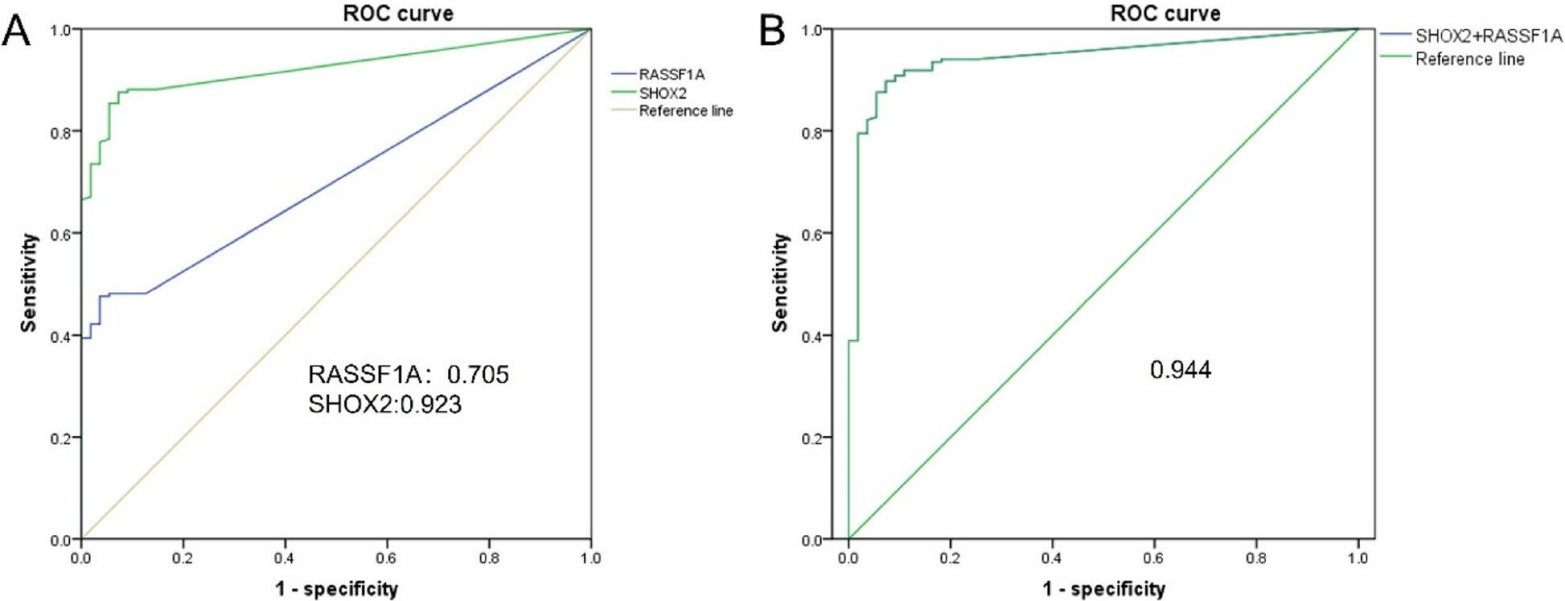
ROC curve determines the cutoff values of SHOX2 and RASSF1A methylation. **A** ROC curves of SHOX2 and RASSF1A methylation in diagnosing lung cancer. **B** ROC curve of combined methylation of SHOX2 and RASSF1A in diagnosing lung cancer

**Fig. 3:**
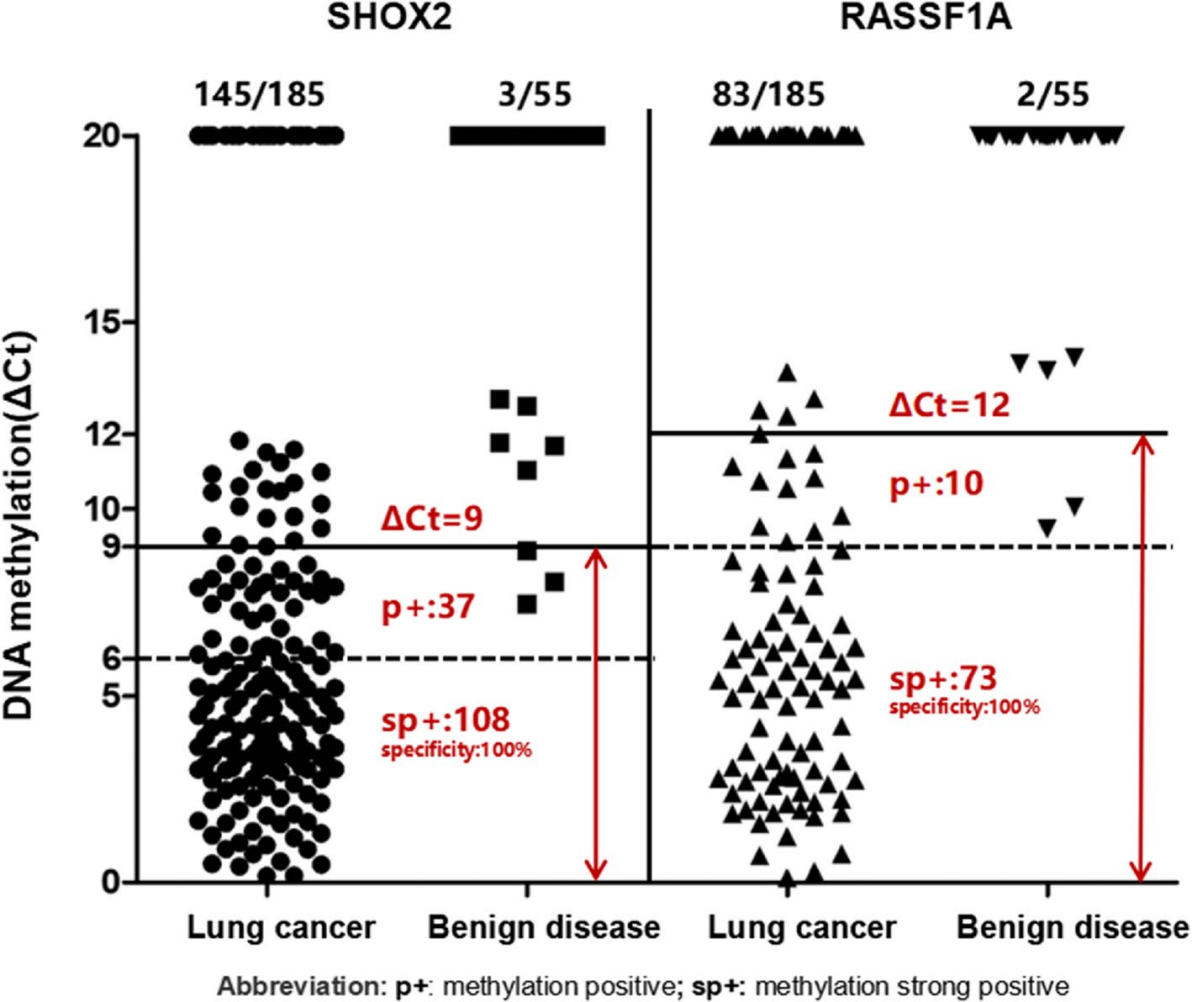
Quantitative analysis of SHOX2 and RASSF1A DNA methylation in lung cancer (*n* = 185) and benign lung disease (*n* = 55) specimens. ΔCt = 20 means Noct

The diagnostic efficacy of SHOX2 and RASSF1A methylation was assessed through ROC curve analysis, revealing the AUC of 0.923 and 0.705, respectively. Additionally, the AUC for LungMe® was determined to be 0.944 (Fig. 2). To assess the diagnostic efficacy, a scatterplot depicting the ΔCt values of SHOX2 and RASSF1A methylation for each sample was generated (Fig. 3). Using a threshold of Ct = 9 for SHOX2, the sensitivity and specificity were determined to be 76.2% and 94.5%, respectively. For RASSF1A, using a ΔCt threshold of 12, sensitivity and specificity were found to be 44.9% and 96.4%, respectively. A smaller ΔCt value indicated a higher concentration of tumor cells in the sample. To achieve a specificity of 100%, we defined strong-positive SHOX2 methylation as ∆Ct_SHOX2_ ≤ 6, and a weak positivity as 6 < ∆Ct_SHOX2_ ≤ 9. Similarly, strong-positive RASSF1A methylation was defined as ΔCt_RASSF1A_ ≤ 9, and a weak-positive RASSF1A methylation was defined as 9 < ΔCt_RASSF1A_ ≤ 12. Based on these definitions, 74.5% (108/145) of positive cases were classified as strong-positive for SHOX2 with 100% specificity, while 88.0% (73/83) of positive cases were classified as strong-positive for RASSF1A with 100% specificity. In summary, SHOX2 and RASSF1A exhibited different diagnostic thresholds, and the appropriate ΔCt thresholds effectively differentiated between lung cancer and benign diseases.

### Gene methylation as an adjunct to cytological and histological diagnosis

The diagnostic efficacy of SHOX2 and RASSF1A methylation, cytology, and histology was analyzed and illustrated in Table 3 across different pathological subgroups, including histology subtype, nodule location, pathological staging, and nodule size. The diagnostic sensitivity of cytology and histology ranged from 20.0%-35.1% and 42.9%-80%, respectively, while the positive detection rate of LungMe® methylation ranged from 70.0% to 100%. Incorporating SHOX2 and RASSF1A DNA methylation as an adjunct to cytological and histological diagnosis significantly improved the diagnostic sensitivity of bronchoscopy, resulting in a combined detection positive rate of 96.8%.

**Table 3:** Diagnostic yield of methylation, cytology, and histology analyses in different subgroups of LC

Tumor Classification		Bronchoscopy					
		Exfoliated cell BALF samples				Biopsy Histology +	Combined results
		SHOX2 +	RASSF1A +	LungMe® +	Cytology +		
		n (%)	n (%)	n (%)	n (%)	n (%)	
Histology subtype							
LUAC	(*n* = 104)	70 (67.3)	48 (46.2)	85 (81.7)	31 (29.8)	60 (57.7)	99 (95.2)
LUSC	(*n* = 53)	49 (92.5)	18 (34.0)	50 (94.3)	16 (30.2)	38 (71.7)	53 (100.0)
SCLC	(*n* = 23)	22 (95.7)	16 (69.6)	22 (95.7)	8 (34.8)	14 (60.9)	22 (95.7)
Metastatic Cancer	(*n* = 5)	4 (80.0)	3 (60.0)	5 (100.0)	1 (20.0)	3 (60.0)	5 (100.0)
Total	(*n* = 185)	145 (78.4)	85 (45.9)	162 (87.6)	56 (30.3)	115 (62.2)	179 (96.8)
Nodule location							
Central	(*n* = 46)	43 (93.5)	23 (50.0)	43 (93.5)	13 (28.3)	27 (58.7)	45 (97.8)
Peripheral	(*n* = 127)	95 (74.8)	55 (43.3)	108 (85.0)	39 (30.7)	82 (64.6)	122 (96.1)
Pathologic staging							
I	(*n* = 28)	22 (78.6)	8 (28.6)	22 (78.6)	7 (25.0)	12 (42.9)	25 (89.3)
II	(*n* = 20)	15 (75.0)	8 (40.0)	17 (85.0)	5 (25.0)	10 (50.0)	19 (95.0)
III	(*n* = 40)	36 (90.0)	18 (45.0)	29 (92.5)	10 (25.0)	29 (72.5)	39 (97.5)
IV	(*n* = 97)	75 (77.3)	50 (51.5)	85 (87.6)	34 (35.1)	64 (66.0)	94 (96.9)
Largest diameter							
0 ~ 10 mm	(*n* = 10)	6 (60.0)	2 (20.0)	7 (70.0)	2 (20.0)	8 (80.0)	8 (80.0)
11 ~ 50 mm	(*n* = 124)	89 (71.8)	51 (41.1)	102 (82.3)	39 (31.5)	79 (63.7)	119 (96.0)
51 ~ 100 mm	(*n* = 29)	27 (93.1)	16 (55.2)	27 (93.1)	10 (34.5)	16 (55.2)	29 (100.0)
101 ~ 300 mm	(*n* = 12)	12 (100.0)	9 (75.0)	12 (100.0)	2 (16.7)	8 (66.7)	12 (100.0)

Compared to RASSF1A, the positive rate of SHOX2 methylation was higher across all lung cancer pathological subgroups. SHOX2 and RASSF1A complemented each other, and the positive rate of combined diagnosis (LungMe®) exceeded that of using a single index. RASSF1A primarily enhanced the diagnostic sensitivity of LUAC, increasing from 67.3% with SHOX2 alone to 81.7% with combined detection. The diagnostic sensitivity of SHOX2 and RASSF1A was higher in central lung cancer compared to peripheral lung cancer (*P* < 0.01). Overall, the positive rates of LungMe® methylation in patients with stage I, II, III, and IV were 78.6%, 85.0%, 92.5%, and 87.6%, respectively. Our data did not indicate a definite trend change in the methylation positive rate with tumor stage. However, the positive rate of methylation in small pulmonary nodules with a diameter between 0–10 mm was significantly lower than that in larger sizes (*P* < 0.01).

**Fig. 4:**
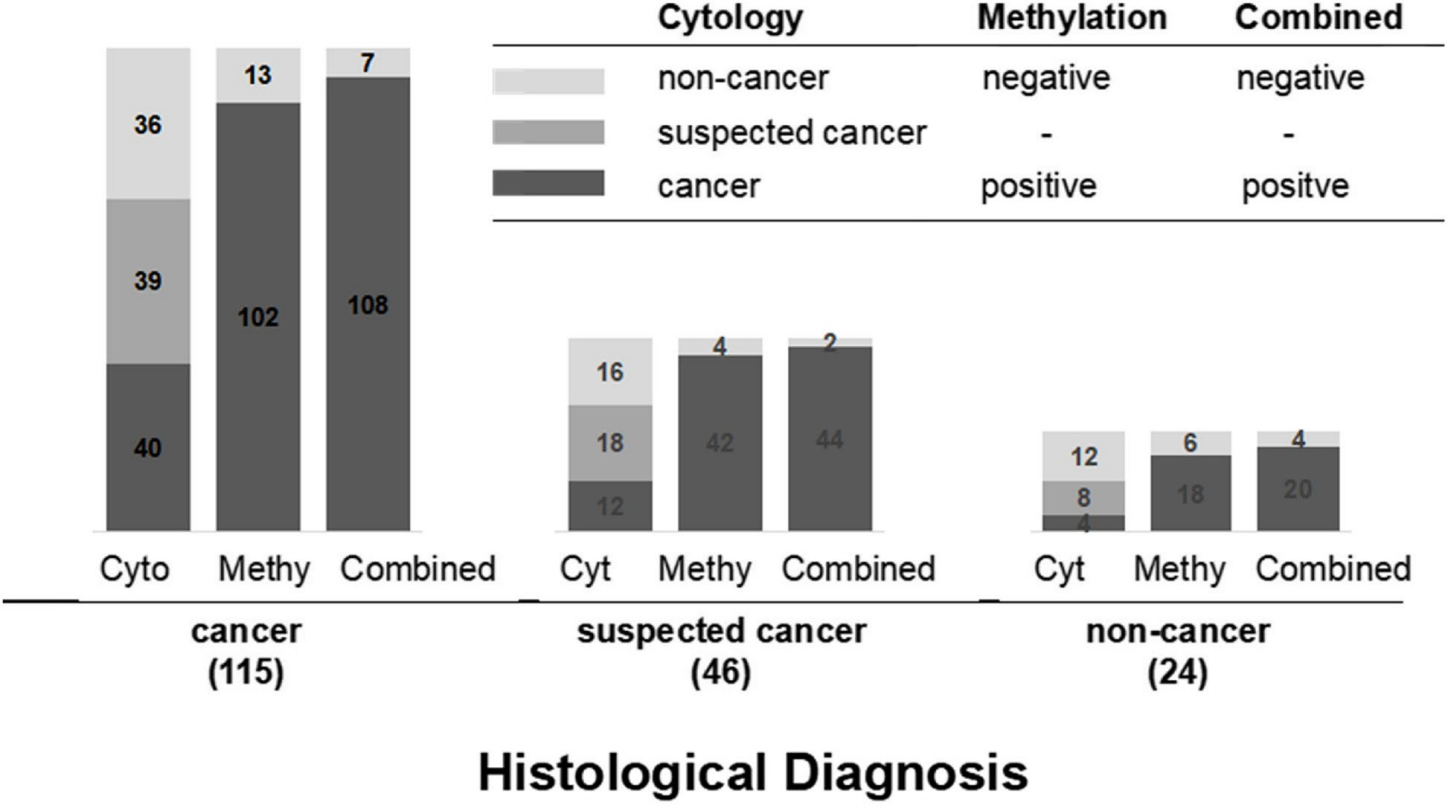
The complementary role of methylation in cytological and histological diagnosis

To demonstrate the complementary role of methylation in cytological and histological diagnosis, we categorized the results of morphological diagnosis into three levels: cancer, suspected cancer, and non-cancer (Fig. 4). Among the histology cancer group, 40 out of 115 exfoliated cell specimens were cytologically diagnosed as “cancer”, while 102 specimens tested positive for methylation. Within the histology suspected-cancer group, 12 out of 46 specimens were cytologically diagnosed as “cancer”, whereas 42 specimens showed methylation positivity. In the histology of non-cancer group, 4 out of 24 specimens were cytologically diagnosed as “cancer” and 18 were found to be methylation positive. Our data demonstrated that when there is suspicion of cancer or non-cancer diagnosis, methylation can serve as a prompt or aid in confirming the diagnosis of malignant lung disease.

### Correlation between different IHC and methylation markers

We conducted IHC analysis of Ki67, Napsin A, TTF-1, CK5/6, P63, and P40 expression in LC samples (Table 4). The medium and high expression of Ki67 group had a higher proportion of patients with SHOX2( +) (*P* < 0.01) compared to those with low Ki67 expression. However, RASSF1A methylation did not exhibit this phenomenon (*P* = 0.35). LUSC (10/33) and SCLC (17/21) patients had a higher incidence of high Ki67 expression compared to LUAC (6/40). We also observed a negative correlation between a lower SHOX2 methylation detection rate and positive Napsin A expression (*P* = 0.0081), as well as a positive correlation between a higher RASSF1A methylation detection rate and positive TTF-1 expression (*P* = 0.0098) and negative P40 expression (*P* = 0.0090). No correlations were found between methylation and other IHC markers. Napsin A showed an 85.1% sensitivity for LUAC and 100% specificity for LUSC and SCLC, while TTF-1 positivity detected 87.0% LUAC, 73.9% SCLC, and 2.3% LUSC. P40 exhibited better performance compared to CK5/6 and P63, with a sensitivity of 94.4% for LUSC and a specificity of 97.0% for LUAC and SCLC.

**Table 4:** Correlation between different IHC and methylation markers

Tumor Classification		SHOX2 +	RASSF1A +	LUAC	LUSC	SCLC	
		n (%)	n (%)	n (%)	n (%)	n (%)	
Ki67 (%)							
≤ 10% (low)	(*n* = 13)	7 (61.5)	6 (46.2)	10 (76.9)	3 (23.1)	0 (0.0)	
10% ~ 60% (medium)	(*n* = 42)	35 (83.3)	16 (38.1)	24 (57.1)	13 (31.0)	4 (9.5)	
≥ 60% (high)	(*n* = 33)	31 (90.9)	18 (54.5)	6 (18.2)	10 (30.3)	17 (51.5)	
CHITEST		*P* = 0.0065	*P* = 0.3508	*P* < 0.0001	*P* = 0.9077	*P* < 0.0001	
NapsinA							
+	(*n* = 57)	39 (68.4)	29 (50.9)	57 (100.0)	0 (0.0)	0 (0.0)	to LUAC SENS:85.1% SPEC:100%
-	(*n* = 57)	51 (89.5)	24 (42.1)	10 (17.5)	30 (52.6)	17 (29.8)	
CHITEST		*P* = 0.0081	*P* = 0.4212	*P* < 0.0001	*P* < 0.0001	*P* < 0.0001	
TTF-1							
+	(*n* = 85)	64 (75.3)	48 (56.5)	67 (78.8)	1 (1.2)	17 (20.0)	to LUAC and SCLC SENS:84.0% SPEC:97.7%
-	(*n* = 59)	50 (84.7)	20 (33.9)	10 (16.9)	43 (72.9)	6 (10.2)	
CHITEST		*P* = 0.1261	*P* = 0.0098	*P* < 0.0001	*P* < 0.0001	*P* = 0.0422	
CK5/6							
+	(*n* = 57)	46 (80.7)	22 (38.6)	13 (22.8)	43 (75.4)	1 (1.8)	to LUSC SENS:95.6% SPEC:79.7%
-	(*n* = 57)	45 (78.9)	28 (49.1)	39 (68.4)	2 (3.5)	16 (28.1)	
CHITEST		*P* = 0.8214	*P* = 0.3355	*P* < 0.0001	*P* < 0.0001	*P* < 0.0001	
P63							
+	(*n* = 31)	27 (87.1)	9 (29.0)	6 (19.4)	25 (80.6)	0 (0.0)	to LUSC SENS:96.2% SPEC:86.7%
-	(*n* = 40)	32 (80.0)	20 (50.0)	27 (67.5)	1 (2.5)	12 (30.0)	
CHITEST		*P* = 0.3944	*P* = 0.0926	*P* = 0.0003	*P* < 0.0001	*P* < 0.0001	
P40							
+	(*n* = 36)	30 (83.3)	12 (33.3)	2 (5.5)	34 (94.4)	0 (0.0)	to LUSC SENS:97.1% SPEC:97.0%
-	(*n* = 70)	54 (77.1)	40 (57.1)	50 (67.5)	5 (7.1)	15 (21.4)	
CHITEST		*P* = 0.2383	*P* = 0.0090	*P* < 0.0001	*P* < 0.0001	*P* < 0.0001	

### Comparison of the detection efficiency of serum tumor markers, pathology, and methylation markers

We assessed serum tumor markers (TMs) levels using chemiluminescent immunoassay detection (Table 5). The results showed that CEA, SCCA, and CYFRA21-1 had a positive detection rate of 48.8%, 26.2%, and 55.8%, respectively. CEA demonstrated higher sensitivity for LUAC (59.2%) and SCLC (45.5%) than for LUSC (30.8%), while SCCA showed higher specificity for LUSC. CYFRA21-1 exhibited good diagnostic sensitivity for various pathological subtypes ranging from 50.0% to 65.4%, with a relatively satisfied specificity (85.2%).

**Table 5:** Comparison of the sensitivity and specificity of serum tumor markers, pathology, and methylation detection for different subtypes of lung cancer

Biomarkers	Sencitivity				Specificity	PPV	NPV
	LC (total)	LUAC	LUSC	SCLC	Benign		
	*n* = 172	*n* = 98	*n* = 52	*n* = 22	*n* = 55		
Serum							
CEA	48.80%	59.20%	30.80%	45.50%	74.10%	85.70%	31.30%
SCCA	26.20%	10.20%	61.50%	13.60%	81.50%	81.80%	26.20%
CYFRA21-1	55.80%	50.00%	65.40%	59.10%	85.20%	92.30%	37.70%
CEA + SCCA + CYFRA21-1	75.00%	73.50%	82.70%	63.60%	55.60%	84.30%	41.10%
Exfoliated cell BALF + Biopsy							
Cytology	30.30%	29.60%	28.80%	36.40%	100.00%	100.00%	29.40%
LungMe®	86.00%	80.60%	92.30%	95.50%	92.70%	97.40%	67.60%
Histology	60.50%	55.10%	71.20%	59.10%	100.00%	100.00%	49.80%
Cytology + LungMe®	91.30%	86.70%	98.10%	95.50%	92.70%	97.40%	76.90%
Histology + Cytology + LungMe®	96.50%	94.90%	100.00%	95.50%	92.70%	98.20%	83.90%

The cutoff values for CEA, SCCA, and CYFRA21–1 were 5 ng/mL, 1.5 ng/mL, and 3.3 ng/mL, respectively

Compared to serum TMs, the methylation analysis of LungMe® in BEC demonstrated the highest diagnostic sensitivity at 86.0%, while cytology and histology had sensitivities of 30.2% and 60.5%, respectively. Combining LungMe® not only significantly improved the sensitivity of pathological diagnosis, but also enhanced its negative prediction value (NPV). Importantly, LungMe® exhibited excellent tumor specificity of 92.7%.

### The comprehensive lung cancer diagnostic work-up, including LumgMe® methylation

**Fig. 5:**
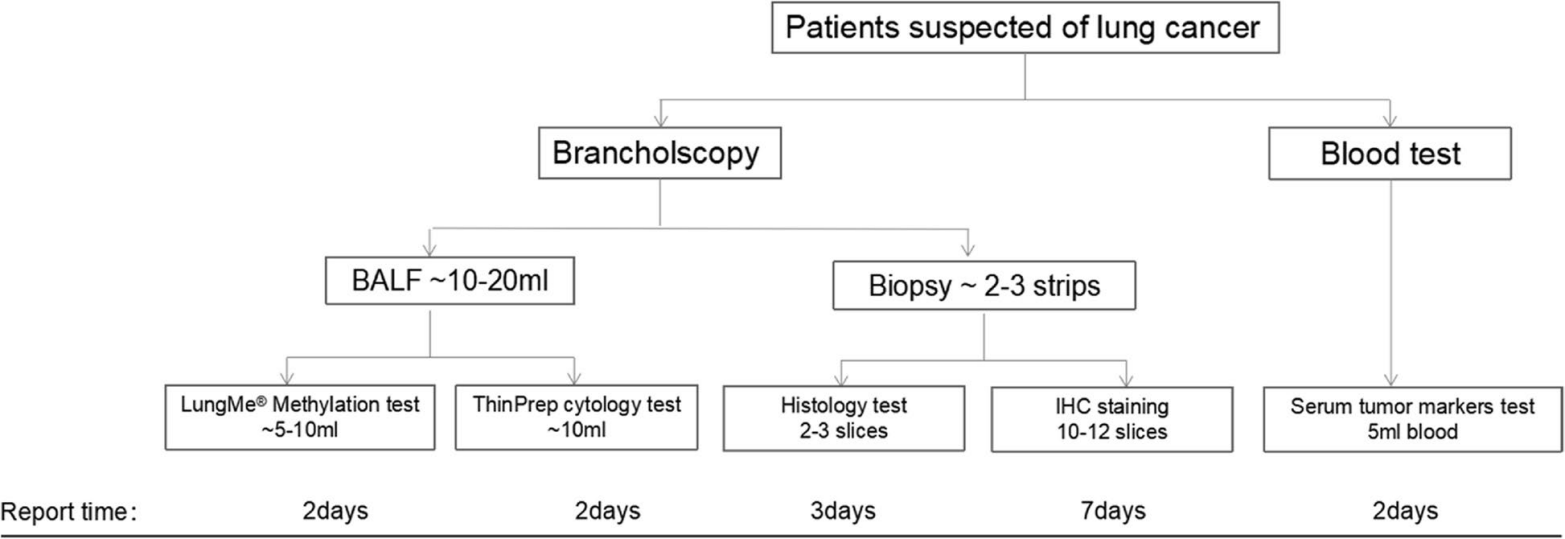
The comprehensive lung cancer diagnostic work-up, including LumgMe® methylation

Figure 5 illustrates the comprehensive lung cancer diagnostic work-up conducted at our hospital. LungMe® methylation testing is performed alongside pathological analysis using sample material routinely obtained during bronchoscopy. Simultaneously, non-invasive detection of blood tumor markers is carried out in the central laboratory.

Case sharing: In this particular case, the inclusion of LungMe® methylation testing, along with cytology and other available clinical information, aided clinicians and pathologists in confirming the presence of malignant disease. This timely diagnosis enabled prompt treatment for the patient.

**Fig. 6:**
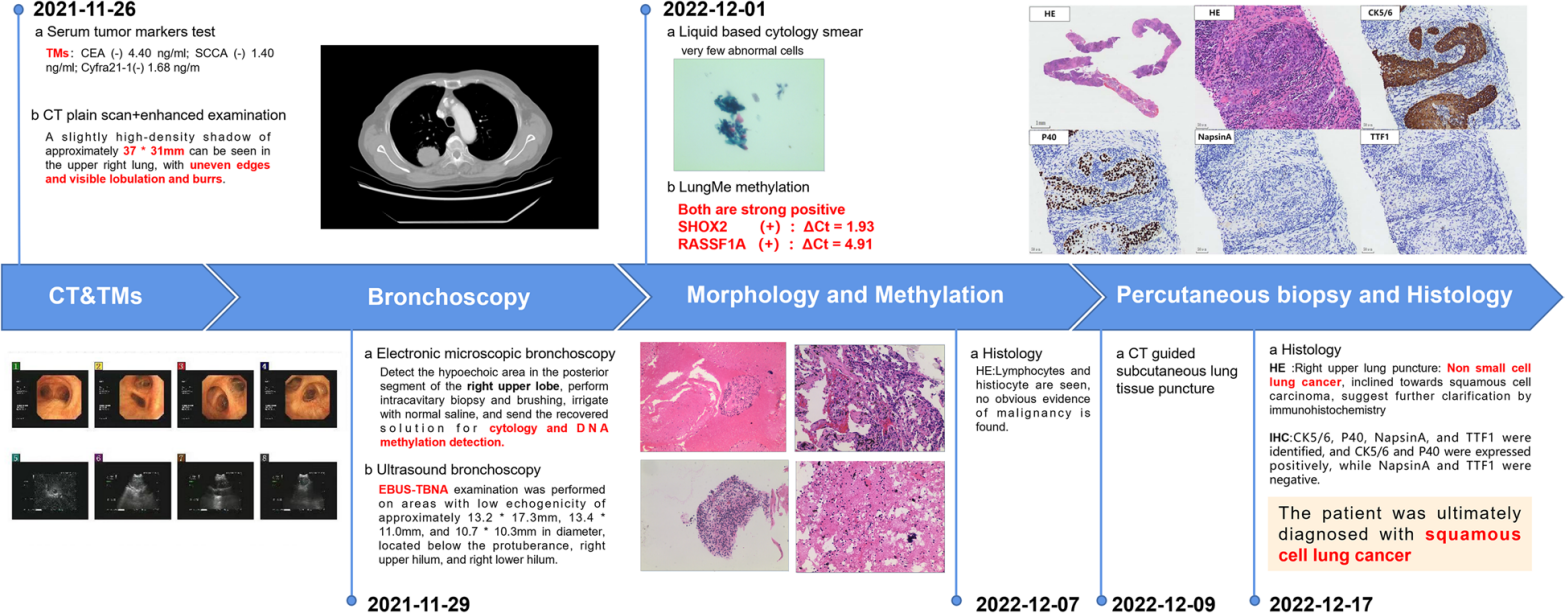
The patient's diagnosis process at the Affiliated Hospital of Nantong University

Patient: A 72-year-old male was admitted to the Affiliated Hospital of Nantong University with “right space-occupying lesions.” CT imaging revealed a grid-like mass with soft tissue density shadow and fuzzy shadow in the right upper lung, measuring approximately 36 × 27 mm. The lesion displayed shallow lobulation and irregular borders. Additionally, grid-like high-density shadow and fuzzy shadow were observed around the lesion, with adjacent pleural involvement. The patient's diagnostic process at our hospital is depicted in Fig. 6.

## Discussion

Lung cancer is a heterogeneous disease, affected by environmental exposures and individual genetic or epigenetic susceptibilities. In 2015, the incidence of lung cancer in China was approximately 787,000, with 66% (520,000) in males and 34% (267,000) in females [17]. The incidence in males gradually declined until 2011 but has recently shown an upward trend. Among females, there has been a rapid increase in lung cancer incidence since 2011. This trend can be attributed to the decreasing smoking rates among males and the implementation of LDCT screening in China since 2011. Histopathological analysis revealed a decrease in the proportion of LUSC and a continuous rise in LUAC from 2002 to 2015, particularly in females [18]. Although the prevalence of smoking among women in China is notably low, there is an emerging group of female non-smokers who are being diagnosed with lung cancer, predominately affected by LUAC. This explains why 96.5% of female lung cancer patients in our study were non-smokers, with 83.6% having LUAC. The proportion of LUAC and LUSC/SCLC in male patients was 40.4% and 59.6% among smokers, compared to 49.3% and 50.7% among non-smokers. The influence of gender on the susceptibility to lung cancer remains substantial, particularly among non-smokers.

LDCT screening improves the detection of early-stage lung cancer, especially peripheral LUAC, as bronchoscopy and transthoracic aspiration face challenges in identifying early, small, peripherally located lesions. Initial BEC cytology and/or biopsy are crucial for confirming an early diagnosis and avoiding unnecessary repeat sampling, complications, and treatment delays. Combining morphology and molecular methylation detection appears to enhance diagnostic accuracy. In our study, we described the integration of this new technology into our routine pathological detection process and discussed how to incorporate different test results for a more comprehensive and definitive pathological diagnosis report.

For a specific detection technology, there is a balance between sensitivity and specificity. Methylation analysis can assist in differentiating various treatment-related lung conditions, such as cancer, pulmonary infection, or tuberculosis. In our study, although the ΔCt method reduced the diagnostic sensitivity from 94.1% to 87.6%, it significantly improved the specificity of methylation detection from 72.7% to 90.9%. ΔCt was deemed to be the more appropriate judgment method for methylation results.

Our results indicated that SHOX2 and RASSF1A in BEC had different threshold values:ΔCt = 9 for SHOX2 and ΔCt = 12 for RASSF1A. This difference is primarily determined by the specificity of each methylation marker and the stability of the FAM channel (used for RASSF1A) compared to the VIC channel (used for SHOX2). When comparing data from FFPE samples [19] and pleural effusion [15], we observed that RASSF1A can maintain a cutoff value of 12 across different sample types, with high specificity ranging from 95.6%-97.9%. On the other hand, for FFPE samples, the threshold for SHOX2 needed to be lowered to 7.5 to achieve a specificity of 92.1%. When working with a new sample type, it is essential to re-evaluate the cutoff value for the marker. Additionally, during bronchoscopic examinations, various cytological specimens can be obtained, such as brushing, selective bronchial lavage, curettage, transbronchial needle aspiration, rinse fluids of the forceps, and all aspirated fluids. Due to the heterogeneity of the sampling, the qualitative methylation detection results of the samples reflect the methylation status of the lesion. However, quantitative ΔCt values are helpful for clinicians to evaluate the positive predictive value of methylation. Our results showed that the positive predictive value of strong-positive methylation is close to 100%. Notably, a very high proportion of cases exhibited strong-positive methylation, namely 74.5% for SHOX2 and 88.0% for RASSF1A.

The sensitivities of biopsy, cytology, and methylation analysis of SHOX2 and RASSF1A in BEC were evaluated during the bronchoscopic examination. Methylation analysis based on nucleic acid amplification techniques exhibited much higher sensitivity (70.0%-100%) than conventional morphological diagnosis (20.0%-80.0%). Additionally, a significant proportion of morphological diagnoses were “suspected cancer” accounting for 24.9% in histology and 35.1% in cytology. Objective and quantitative methylation results can prompt or assist pathologists in confirming the diagnosis of malignant lung cancer. In this study, we demonstrated that the combination of these three techniques could increase the diagnostic rate to 96.8%. Furthermore, 48 out of 185 patients diagnosed with lung cancer (25.9%) received a positive diagnosis only through methylation analysis. Consistent with previous studies [14, 20], SHOX2 proved to be a highly sensitive indicator for the diagnosis of lung cancer, with higher levels of methylation observed in patients with SCLC (95.7%) and LUSC (92.5%) compared to patients with LUAC (67.3%). The addition of RASSF1A increased the detection rate for LUAC from 67.3% to 81.7%. Notably, the sensitivity of RASSF1A in diagnosing LUSC (34.0%) was significantly lower than that for LUAC (46.2%) and SCLC (69.6%) (*P* < 0.01). Our study also revealed differences in diagnostic rates based on the nodule location, pathological staging, and nodule size, although these differences were not statistically significant. This may be due to the high sensitivity of methylation analysis as well as the limited number of samples.

Subsequently, the correlation between SHOX2, RASSF1A methylation, and various immunohistochemical markers was analyzed. Our findings revealed a positive correlation between the expression of Ki-67 and the methylation level of SHOX2, but not RASSF1A. Similar findings were reported by Gao, et al*.* (2022) [16], who suggested that high Ki-67 expression, in line with positive methylation, may be associated with rapid tumor progression and the need for aggressive treatments, even in cases diagnosed as early-stage lung cancer. Additionally, patients who tested negative in the combined methylation assay may have lesions with relatively slow tumor cell proliferation and good cancer-free survival rates. Furthermore, five other IHC markers were evaluated, including LUAC-specific Napsin A and TTF-1, LUSC-specific CK5/6, P63, and P40. We observed that a lower detection rate of SHOX2 methylation was correlated with positive Napsin A expression (*P* = 0.0081), while a higher RASSF1A methylation detection rate was correlated with a positive TTF-1 expression (*P* = 0.0098) and a negative P40 expression (*P* = 0.0090), both indicating a diagnosis of non-LUSC. This observation aligns with the impact of histological lung cancer subtypes on SHOX2 and RASSF1A methylation levels. The sensitivity of SHOX2 in diagnosing LUSC is comparatively limited, whereas patients with LUSC exhibit lower levels of RASSF1A methylation compared to patients with LUAC and SCLC. No correlation was observed between methylation and other IHC markers. In addition to sensitivity, Napsin A, TTF-1, and P40 exhibit superior specificity compared to CK5/6 and P63.

Over the years, various antigens found in blood have been assessed as potential biomarkers for lung cancer. The most extensively studied biomarkers include carcinoembryonic antigen (CEA), squamous cell carcinoma antigen (SCCA), and CYFRA21-1. Although serologic biomarkers can be conveniently and economically analyzed, their sensitivity in diagnosing early tumors is insufficient, and their specificity for benign lesions is moderate. Our study observed variations in the sensitivity and specificity of each tumor marker (TMs) across the different types of lung cancer. Therefore, it appears that a single unique antigen biomarker is not valuable for diagnosis, and a multi-antigen approach should be considered. However, this approach may lead to a rapid decrease in specificity. Efforts have been made to use ctDNA methylation in plasma as a diagnostic and screening biomarker to determine whether nodules identified by LDCT are benign or malignant [21]. The results demonstrated that, at a fixed specificity of 90%, the sensitivity of SHOX2 and PTGER4 methylation in plasma for lung cancer was 67%. Similarly, at a fixed sensitivity of 90%, the specificity was 73%. There is currently a debate regarding the use of ctDNA for early cancer detection due to the small tumor burden and low mutant allele fraction (MAF). When tumors have a diameter of 10–15 mm or smaller, their MAF is approximately 0.01% (one tumor DNA molecule admixed with 10,000 normal DNA molecules). The use of 10 mL of blood (4 mL of plasma) will likely contain less than a complete cancer genome, rendering the diagnosis of cancer impossible. Recent data from Grail confirm the low sensitivity for early cancer detection (< 30% for Stage I-II tumors and < 20% for Stage I tumors), but specificity was high at 99.5% [22, 23]. Apart from tumor size, detectable ctDNA levels in plasma are influenced by factors such as tumor mitotic volume and metabolic activity for breast and lung cancer, the surface area of tumors invading beyond the subserosa for colorectal cancer, and so on [23]. Instead of early tumor detection, serum TMs and ctDNA methylation in plasma may play a more crucial role in monitoring the treatment efficacy and recurrence in advanced stages.

Finally, a comprehensive diagnostic evaluation for lung cancer that was conducted at our hospital was presented. The LungMe® test can be performed in conjunction with routine pathological analysis of sample material obtained during bronchoscopy. In our study, it was found that in up to 30% of patients with suspected lung cancer, clinicians and pathologists were unable to confirm a diagnosis of malignant lung disease, even with cytological and histological analyses of materials collected during bronchoscopy. However, a case study demonstrated that the addition of the LungMe® methylation test, in conjunction with cytology and other available clinical information, aided clinicians and pathologists in corroborating the existence of malignant disease. Consequently, this expedited the identification of the ailment and the administration of suitable therapeutic interventions for the patient. Serving as a powerful complement and extension to conventional methods, the combined analysis of SHOX2 and RASSF1A methylation could enhance the accuracy of lung cancer diagnosis, offering satisfactory sensitivity and specificity.

The novelty of this study lies in the incorporation of methylation, cytology, and histological diagnosis as routine tests in hospitals. The study aims to explore the synergistic utilization of these indicators to reinforce and validate one another, ultimately leading to a comprehensive diagnosis that is characterized by enhanced accuracy and clarity. The study has certain limitations that merit consideration. While SHOX2 and RASSF1A serve as promising cancer biomarkers and exhibit commendable diagnostic capabilities for detecting lung cancer in alveolar lavage fluid, they cannot discern the pathological subtypes. SHOX2 and RASSF1A are not suitable for tissue tracing if it is applied to pleural fluid or blood samples, as they are also positive for breast cancer, gastric cancer, and esophagus cancer. The primary function of SHOX2 and RASSF1A is to facilitate the differentiation between benign and malignant. SHOX2 and RASSF1A methylation detection is most effectively employed with tissue-specific samples such as bronchoalveolar lavage fluid or sputum.

Additionally, the research primarily centers on evaluating the differential diagnostic potential of methylation in distinguishing between benign and malignant tumors, yet it fails to capture the dynamic changes in the stage progression of tumor lesions. Methylation exhibits a relatively high positive rate in the early stages of tumors. Relying solely on the positive rate of methylation may not provide a clear indication of the pathological stage. Furthermore, the study overlooks the nuances of various sampling methods and concentrates solely on scrutinizing disparities in methylation, cytology, and histology within a single bronchoscopy sample.

## Data Availability

All dataset generated or analysed during the current study are included in the article and further inquiries can be directed to the corresponding author/s ".

## Abbreviations

BEC: Broncho-exfoliated cells
LDCT: Low-dose chest computed tomography
BALF: Bronchial or bronchoalveolar lavage fluid
TCT: ThinPrep cytology test
LUAC: Lung adenocarcinoma
LUSC: Lung squamous cell carcinoma
SCLC: Small-cell lung carcinoma
ROC: Receiver operating characteristic curve
AUC: Area under the curve
